# Bibliographical Department

**Published:** 1881-10

**Authors:** 


### BIBLIOGRAPHICAL DEPARTMENT.

"Nothing extenuate nor set down aught in malice."

*Anatomical Studies upon Brains of Criminals. A contribution to Anthropology, Medicine, Jurisprudence, and Psychology. By Moriz Benedikt. Translated from the German by E. P. Fowler, M. D. Wm. Wood & Co., New York, Publishers.* Since the elaborate experiments of Hitzig and Ferrier on the localization of cerebral function, a most decided impetus has been given to the study of brain physiology and pathology, with the result of opening many questions pertaining to medicine, jurisprudence, and psychology. This last contribution of Professor Benedikt is an effort to throw light upon the moral and legal status of criminals by a careful study of the brains of well known violators of both moral and civil laws. Twenty-two cases serve as the material from which his deductions are drawn, a number hardly sufficient to more than *suggest* further labor in what promises to be a rich field. Variations in the size of the cerebellum and in the situation of the cerebral fissures, together with reversions of type in the arrangement of convolutions, and fissures with unusual connections, which in many cases resemble the brains of the lower animals, are noted. The brains of murderers would seem to suggest type reversions to that of the bear; whilst robbers with feeble intellects, or with cunning and knavery well developed, more closely *resemble* the arrangement of convolutions, fissures, etc., found in the fox. Before much practical value can be attached to Professor Benedikt's discovery, more uniformity of results, more observations and comparisons upon criminal and non-criminal brains of the *same race* (so as to eliminate all *race* peculiarities) will be requisite. Moreover, such lesions or abnormalities as are found should be common to similar criminals in other races. The laws of heredity lend some support to Benedikt's work, in that moral and immoral predispositions, as well as physical and mental characteristics, are capable of being transmitted. The work has been well translated by Dr. E. P. Fowler, and will be read with interest by students of physiology and psychology.

*Landmarks—Medical and Surgical. By Luther Holden. Henry C. Lea's Son & Co., Philadelphia, Publishers.* The third edition of this most valuable little work is now before the public, and should be in the possession of every medical student in the world. Its aim is to teach anatomy *clinically*, and to bring to the aid of the practitioner such help as can be derived from a careful study of anatomy. In the performance of this object Mr. Holden has been most successful. "Landmarks" has been appended to the last edition of Gray's text book on Anatomy.

*The Compend of Anatomy. By Jno. B. Roberts, A. M., M. D.*, is a most excellent little manual of 198 pages "for use in the dissecting room, and in preparing for examinations." A second revised edition, within a few months after the publication of the first, is evidence of its appreciative merit. It is handsomely printed on fine paper, with flexible cloth backs, by C. C. Roberts & Co., 1118 Arch St., Philadelphia ; price, $1.25.

*The North American Review's* contents for October cannot fail to arrest the attention of all readers. The topics discussed are of the highest present interest, and nearly all of the authors are eminent American Statesmen, publicists and litterateurs.

*The Scientific News* is another welcome exchange received at our sanctum, published monthly by Messrs. Munn & Co., 37 Park Row, N. Y., for the small sum of $1.50 per annum. It is handsomely illustrated, printed on fine paper, and has intrinsic worth to all. It is only necessary to add that it emanates from the office of the *Scientific American and Scientific American Supplement*, which weekly publications are standard authority.

*The Gardener's Monthly and Horticulturist* is a very interesting and valuable journal edited by Thomas Meehan, 814 Chestnut street,

Philadelphia, at $2.10 per annum. We are pleased to find it on our exchange table.

*May Iodide of Potassium Excite Bright's Disease? By I. Edmondson Atkinson, M. D. of Baltimore.* This reprint from the *American Journal of the Medical Sciences,* is a paper read at the meeting of the American Medical Association at Richmond, and is of much importance to the physician. Prof. Atkinson treats his subject with ability.

*Chronic Pelvic Abscess:* A contribution to the Differential Diagnosis of abdominal Tumors, by A. F. Erich, M. D., Prof. of Diseases of Women, Coll.of Physicians and Surgeons,Balto., Surgeon in Charge of the Maryland Woman's Hospital, &c.
*Reprint pp.* 6.

We have received and placed upon our exchange list the following foreign publications, since our last issue:

L'ODONTOLOGIE, Vol. I, No. 3. Edited by Dr. Aubeau, Paris. This number contains articles by the Editor and Prof. Pillette; EL MEDICO Y CIRUJANO CENTRO-AMERICANO, edited by Dr. Ferd. C. Valentine, Guatemala, Central America, and the BULLETIN HEBDOMADAIRE of the Board of Health of the City of Brussels, for the week ending August 15th, 1881.

*The Kansas City Dental College* sends us its first annual announcement for session 1881–82. "Being the Dental Department of Kansas City Medical College," we wish it success with a useful career.

*Philadelphia School of Anatomy.* Forty-third annual announcement of the course of lectures received. This institution is too old and favorably known to be commended by us.

*The Baltimore Medical College Announcement* received. We have given a synopsis of this new organization and deem it only necessary to add that its first session is to begin October 1st, 1881, and close in March following.

